# Cooking methods and kitchen ventilation availability, usage, perceived performance and potential in Canadian homes

**DOI:** 10.1038/s41370-023-00543-z

**Published:** 2023-04-15

**Authors:** Liu Sun, Brett C. Singer

**Affiliations:** 1 Air Sectors Assessment and Exposure Science Division, Water and Air Quality Bureau, Health Canada, Ottawa, ON Canada; 2 Indoor Environment Group, Sustainable Energy and Environmental Systems Department, Energy Technologies Area, Lawrence Berkeley National Laboratory, Berkeley, CA USA

**Keywords:** Inhalation exposure, Indoor air quality, Healthy buildings, Cooking pollutants, Range hood, Extractor hood

## Abstract

**Background:**

Cooking is a substantial contributor to air pollutant exposures in many residences. Effective use of kitchen ventilation can mitigate exposure; however, information on its availability, usage, and potential to increase its use across the population has been limited.

**Objective:**

This study aimed to obtain nationally representative information on cooking methods, kitchen ventilation availability and usage, and the potential for education to increase effective usage.

**Methods:**

An online survey was sent to a representative sample of Canadian homes to collect data on cooking methods, the presence and use of mechanical kitchen ventilation devices, perceived device performance, and willingness to implement mitigation strategies. Responses were weighted to match key demographic factors and analyzed using non-parametric statistics.

**Results:**

Among the 4500 respondents, 90% had mechanical ventilation devices over the cooktop (66% of which were vented to the outside), and 30% reported regularly using their devices. Devices were used most often for deep-frying, followed by stir-frying, sautéing or pan-frying, indoor grilling, boiling or steaming. Almost half reported rarely or never using their ventilation devices during baking or oven self-cleaning. Only 10% were fully satisfied with their devices. More frequent use was associated with the device being vented to the outdoors, having more than two speed settings, quiet operation if only one speed, covering over half of the cooktop, and higher perceived effectiveness. After being informed of the benefits of kitchen ventilation, 64% indicated they would consider using their devices more often, preferentially using back burners with ventilation, and/or using higher ventilation device settings when needed.

**Impact:**

This study provides population-representative data on the most used cooking methods, kitchen ventilation availability and usage, and influencing factors in Canadian homes. Such data are needed for exposure assessments and evaluating the potential to mitigate cooking-related pollutant exposures via more effective use of kitchen ventilation. The data can be reasonably extrapolated to the United States, given the similarities in residential construction practices and cultural norms between the two countries.

## Introduction

Cooking has a significant impact on indoor air quality (IAQ). Cooking can release large amounts of particulate matter in all size ranges (ultrafine, fine, and coarse) and many potentially hazardous chemicals in the condensed (particle) and gas phases from the heating of oil, fat, and other food ingredients. Cooking appliances that rely on combustion also release nitrogen oxides (NO_x_) and carbon monoxide (CO) in quantities that can substantially impact IAQ and exceed hazard thresholds [1–6]. The high number and mass concentrations of particles emitted from cooking have been widely reported [7–14]. Chemical pollutants emitted during cooking include organic compounds such as polycyclic aromatic hydrocarbons, carbonyl compounds (e.g., acrolein, formaldehyde, acetaldehyde), elemental carbon, organic carbon, inorganic elements, and particle-bound water-soluble ions [9, 15–22]. Many factors impact the emissions of these pollutants, including cooking method, food ingredient, type of oil, cooking temperature, and fuel type [19, 23–28]. Exposure to pollutants from cooking may have harmful impacts on the respiratory and nervous systems, causing oxidative stress, inflammatory response, and DNA damage [29–31]. Epidemiological studies have demonstrated positive associations between exposure to cooking fumes and respiratory diseases, cardiovascular diseases, and lung cancer [32–37].

The most universally applicable means to reduce exposure to cooking-related pollutants is via the use of effective kitchen ventilation. A ventilation device placed over the cooktop—a range hood or over-the-range (OTR) microwave with an exhaust fan ducted to the outside—is the most efficient mitigation approach, as it can capture pollutants from cooking and cooking burners and remove them before they mix into the occupied space. Many studies have evaluated the performance of range hoods and OTR microwaves and identified factors that influence their effectiveness, including whether venting to the outside, fan flow rate, operation time, hood capture volume, installation height, and cooktop front burner coverage [6, 22, 24, 38–48]. Though less efficient, an exhaust fan located in the kitchen area can also remove pollutants and reduce exposure for home occupants in other rooms, and the ASHRAE residential ventilation standard (62.2) specifies that kitchen exhaust fans must have higher airflows than ducted range hoods [49]. Other approaches to mitigate exposure include increasing ventilation through window opening, use of air cleaning devices, shifting from combustion burners to electric and especially induction, and reducing or avoiding frying and high-temperature broiling or increasing ventilation when doing so.

Several studies have reported occupants’ ventilation behaviors during cooking and some of them have investigated the associations between ventilation behaviors and influential factors (e.g., cooking method, frequency and duration, whether the device is vented to the outside) [22, 47, 48, 50–54]. While these studies provide important insights, none have reported data from a population-representative sample in any country or region.

With the goal of obtaining information needed to assess the hazards associated with cooking-related pollutants and the current availability and use of kitchen ventilation as a mitigation, we developed and implemented a nationwide survey on cooking and kitchen ventilation behaviors in Canadian homes. The specific objectives were to obtain quantitative information from a representative sample on (1) the prevalence of cooking methods that are known to present different emission patterns, (2) the presence of various kitchen ventilation devices in homes, (3) kitchen ventilation device usage, (4) perceived effectiveness and satisfaction with currently used kitchen ventilation devices, (5) relationships among device characteristics, usage, and perceived performance, and (6) the potential to increase the use of mitigation strategies for reducing cooking-related pollutants through education and awareness.

## Methods

### Questionnaire design

A questionnaire, consisting of 42 multiple choice and matrix questions, was developed to collect data on the following five aspects: (1) contextual information about the dwelling (type, size, year of construction, how the kitchen is connected to other rooms) and household demographics (age, gender, province, family size, rental vs. owner-occupied, and household income), (2) cooking (fuel type, cooking device use frequency, the most used cooking methods by meal, and cooktop burner use), (3) mechanical kitchen ventilation device characteristics (presence, type, venting or recirculating, number of speed settings, perceived loudness, and perceived effectiveness), (4) device use behaviors (use frequency, reasons for use and non-use, and frequency of filter cleaning or replacement), and (5) awareness of need and willingness to employ three ventilation strategies (use the device more often, use cooktop back burners while using the device, and use higher speeds when needed). The questionnaire was provided in both English and French. A copy of the questionnaire in English is provided in the supplemental information (SI).

### Survey implementation

Respondents aged 18 years and older were recruited via email through Leger Opinion panel, which is the largest Canadian panel with over 400, 000 members from all regions of Canada. To obtain a representative sample, email invitations were sent to a pool that matched the demographics of the 2016 National Census across age, gender, province, and household income. A unique identification number for each participant was used to ensure no personal information was recorded. Participants who completed the survey received LEO points that are redeemable towards cash, gift cards, Air Miles, or Aeroplan Miles. Prior to a full launch of the survey, a one-week soft launch was undertaken to test for questionnaire issues. Recruitment took place between January 23 and February 24, 2020, and the survey was available for completion during this period.

Procedures for data quality assurance included a validated opt-in process to prevent fake email addresses and get more engaged survey respondents, as well as removing responses from suspicious email addresses, domain names, or IP addresses. To ensure the completeness of the questionnaires, the survey was displayed one question at a time, and participants had to answer all questions before they could submit the questionnaire.

### Statistical analysis

We used all complete survey responses for analysis and compared the respondents’ demographics to the 2016 Canadian Census population to ensure they were representative. If the difference was >5%, a sample weight was developed in a sequential manner for each characteristic that requires adjustment to correct for over- or under-sampling. First, an initial sample weight was computed by taking the ratio of the population proportion to the sample proportion for a certain characteristic that needed adjustment. If an additional characteristic needed to be adjusted, a new weight was computed using the weighted samples obtained from the previous step. This new weight was calculated by dividing the population proportion by the weighted sample proportion. These steps were repeated until all key demographic characteristics (household income, house type, rental vs. owner occupancy, region, age, and gender) matched the population distribution within 5%. All the samples were weighted (with a weight of one if no adjustment is needed) and the statistical analyses were performed on weighted samples.

All analysis was conducted using SAS Enterprise Guide 7.1 (SAS Institute Inc., Cary, NC, USA). Responses to individual survey questions were summarized using estimated population percentage and standard error (provided by PROC SURVEYFREQ). Associations between ventilation behaviors and potentially influential factors were assessed using Rao-Scott design-adjusted chi-square test, at a significance level of 0.05. Results with significant association were further assessed using Cramer’s V test for the strength of association. A value of Cramer’s V >0.15 was considered practically meaningful [55].

## Results

### Survey response population

A total of 4500 complete survey responses were collected. This sample size had a margin of error of ±1.5%, at a 95% confidence level for the overall survey. The margin of error for individual questions where a subgroup of the sample is considered was higher, but no more than ±4.7% (i.e., the estimated population response rate was 4.7% above or below the sample response rate with a 95% probability). These precision bounds assume no significant biases in the respondents relative to the entire population.

Despite efforts to recruit a representative sample across all key demographic characteristics, there was some degree of over or under-sampling of socioeconomic groups that required adjustments. In comparison with the 2016 Census, people who were low-income ($19,999 and under), lived in a rented home, or lived in an owned attached house or apartment were over-represented by 4–15%; while people who had an annual income above $60,000 or lived in an owned or detached house were under-represented by 5–12%. Therefore, adjustments have been applied to household income, house type, rental vs. owner occupancy, region, age, and gender. Table 1 shows the raw and adjusted survey response distribution by demographic and housing characteristics. Figure S1 shows the percentage of survey respondents and the 2016 Canadian Census population by province.

**Table 1:** The raw and adjusted distribution of respondents by demographic and housing characteristics.

Characteristics	Survey response population		2016 Census population%
	Raw% (N)	Adjusted%	
Gender			
Female	50 (2251)	50	51
Male	50 (2249)	50	49
Age			
18–24	11 (495)	11	9
25–34	16 (717)	17	13
35–44	17 (765)	17	13
45–54	20 (903)	18	14
55–64	17 (765)	17	14
65 and older	19 (855)	20	17
Region			
Ontario	38 (1710)	38	38
Quebec	23 (1036)	24	23
Prairies (AB, SK, MB)	18 (809)	18	19
British Columbia	14 (630)	13	13
Atlantic (NB, NL, NS, PEI)	7 (315)	7	7
Total annual household income (CAD)			
$19,999 and under	20 (877)	9	10
$20,000–$39,999	22 (989)	16	17
$40,000–$59,999	21 (923)	16	16
$60,000–$99,999	17 (781)	24	25
$100,000–$149,999	10 (431)	16	18
$150,000 and over	5 (235)	13	15
Prefer not to answer	6 (264)	6	–
Dwelling type			
Detached house	45 (2019)	55	54
Attached house^a^	16 (704)	12	12
Apartment	36 (1603)	31	34
Mobile home	3 (127)	1	1
Other	1 (47)	1	–
Do you own your home			
Yes	53 (2395)	66	68
No (e.g. rented)	47 (2105)	34	32
Home construction year			
Before 1900	3 (119)	2	–
1900–1940	6 (275)	6	–
1941–1960	11 (490)	10	–
1961–1980	24 (1079)	23	–
1981–2000	21 (954)	24	–
After 2000	21 (932)	25	–
Do not know	15 (651)	12	–
Home size (m^2^)			
93 and under	24 (1058)	20	–
94–186	38 (1727)	40	–
187–279	13 (597)	17	–
280 and over	4 (169)	6	–
Do not know	21 (949)	18	–
Number of residents			
1–2	63 (2827)	59	63
3–4	28 (1274)	32	29
5 and over	9 (399)	9	8
How the kitchen is connected to other parts of your home			
No walls separating the kitchen from the living and dining room (open concept kitchen)	54 (2452)	57	–
Separate room with open doorways	38 (1720)	37	–
Separate room with a door(s) that can be closed	5 (208)	5	–
Other	1 (48)	1	–
Do not know	2 (72)	1	–

*AB* Alberta, *SK* Saskatchewan, *MB* Manitoba, *NB* New Brunswick, *NL* Newfoundland, *NS* Nova Scotia, *PEI* Prince Edward Island.

^a^Attached house includes townhouse, row house, and semi-detached house.

### Characteristics of cooking

Most results in this section and subsequently are presented with simple language that treats responses as accurate. More precise language is used for parameters thought potentially or shown previously to be subject to recall or self-reporting biases, for example, how often a range hood is used when cooking [48].

The majority of respondents had electric cooktops (86%) and ovens (91%) with almost all others having natural gas appliances. The breakdown of cooking fuels was similar to that reported in the Canadian Human Activity Pattern Survey 2, where 84% of respondents used electric and 16% used natural gas cooktops [56]. The percentage of households using natural gas for cooking was much lower in comparison to the U.S. (38%) [57]. Less than 1.5% of respondents reported using other fuels for cooking, including propane, butane, and wood.

**Fig. 1:**
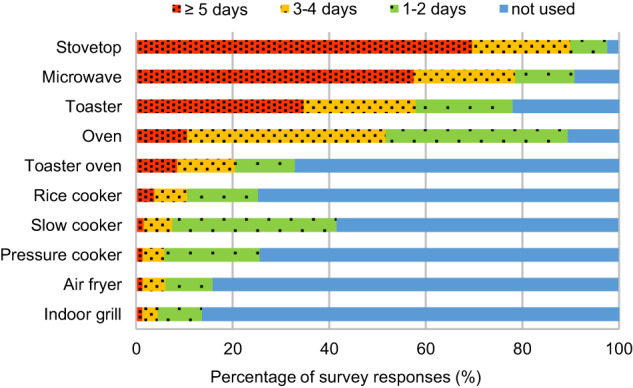
Frequency of cooking appliance use during a typical week. The results were based on 4500 survey responses. The frequency categories are more than five days a week (red), three to four days a week (yellow), one to two days a week (green), and not used (blue).

The top four cooking appliances that participants reported using at least three days a week were cooktop (by 90% of respondents), microwave (79%), toaster (58%), and oven (52%) (Fig. 1). The most frequently reported cooking methods were toasting for breakfast (52%), microwave use for lunch (44%), and baking for dinner (53%) (Figure S2). Sautéing or pan-frying was the second most frequent method for all three meals. Similar to the trend reported by Sun et al. [47], from breakfast to dinner, the frequencies of toaster use declined while baking and boiling increased dramatically.

More than half of the respondents (58%) indicated that they prefer using the cooktop front burners, 31% used both burners equally, and 11% used the back burners more often. About half of the respondents reported differences in cooktop or oven use by season, with 43% indicating that they used these appliances more frequently during winter than other seasons.

### Characteristics of kitchen venting/recirculating devices

Most respondents had a range hood (59% under-cabinet, 7% wall-mounted, 3% ceiling-mounted) or OTR microwave (19%), with two-thirds reporting that the device was vented to the outdoors (66% of range hoods and 67% of OTR microwaves). Two percent of respondents had downdrafts, with 80% of those being vented to the outside. Ten percent reported having no device above or at the cooktop, and 0.5% reported having other types of devices (e.g., wall exhaust). For homes with a gas or propane stove (14% of total), 74% had a vented range hood, and for homes with an electric stove (86% of total), 64% had a vented range hood.

Home size, construction year, and renter vs. owner occupancy each had a large influence on the likelihood of device presence. Large fractions of respondents who reported not having any device lived in homes smaller than 186 m^2^ (86%), rental units (57%), or buildings built before 1960 (52%). None of the identified building or household factors was strongly associated with whether devices vented to the outside or only recirculated the air. Some of the analyses presented below consider the venting and recirculating devices together and some explore venting as a potential explanatory factor.

Only 23% of range hoods and 11% of OTR microwaves fully covered the cooktop front burners. Most range hoods (69%) and OTR microwaves (76%) covered only part of the cooktop front burners and about 10% had front burners that were mostly not covered by the hood.

**Fig. 2:**
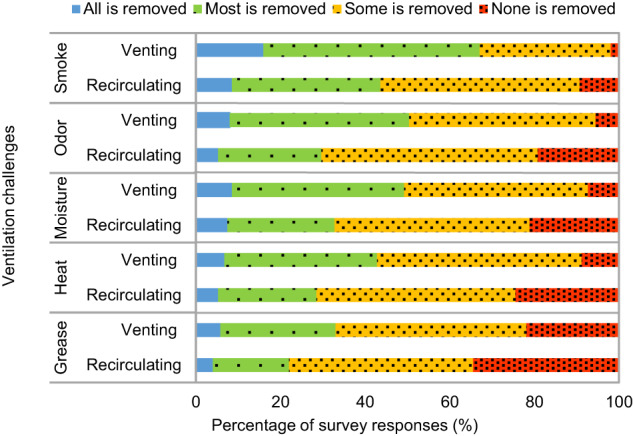
Perceived effectiveness of venting and recirculating devices for specific challenges. Results presented for 2439 respondents with a venting device and 1056 with a recirculating device. The ventilation challenges are categorized by the level of removal achieved: all is removed (blue), most is removed (green), some is removed (yellow), and none is removed (red).

Performance was perceived to be better for venting over recirculating devices in all aspects, including removal of smoke (Cramer’s V = 0.25), odor (Cramer’s V = 0.25), moisture (Cramer’s V = 0.22), and heat (Cramer’s V = 0.22), as well as grease capture (Cramer’s V = 0.16) (Fig. 2). Given the fundamental differences in how the two types of devices operate, the moderate differences in perceived effectiveness are noteworthy. Irrespective of venting, perceived effectiveness was not strongly different among range hoods, OTR microwaves, and downdrafts, suggesting device ventilation mode had a stronger impact on perceived performance than device type.

Only 10% of respondents who had a venting device and 7% of respondents who had a recirculating device were fully satisfied with their device. Respondents highlighted the following aspects to be improved: less noisy (60% of venting and 64% of recirculating devices), better odor removal (42% of venting and 49% of recirculating devices), better grease removal (41% of venting and 47% of recirculating devices), better smoke removal (38% of venting and 50% of recirculating devices), easier to clean grease screens (35% of venting and 33% of recirculating devices), more coverage for front burners (27% of venting and 25% of recirculating devices), and better heat removal (20% of venting and 32% of recirculating devices).

### Device usage and maintenance

Among the reasons noted for using a device, removing smoke (79%) and odors (60%) were the most common. Other reasons included removing moisture (38%), capturing grease (27%), removing heat (23%), removing air pollutants emitted by cooking (23%), general kitchen ventilation (23%), and others (1%).

**Fig. 3:**
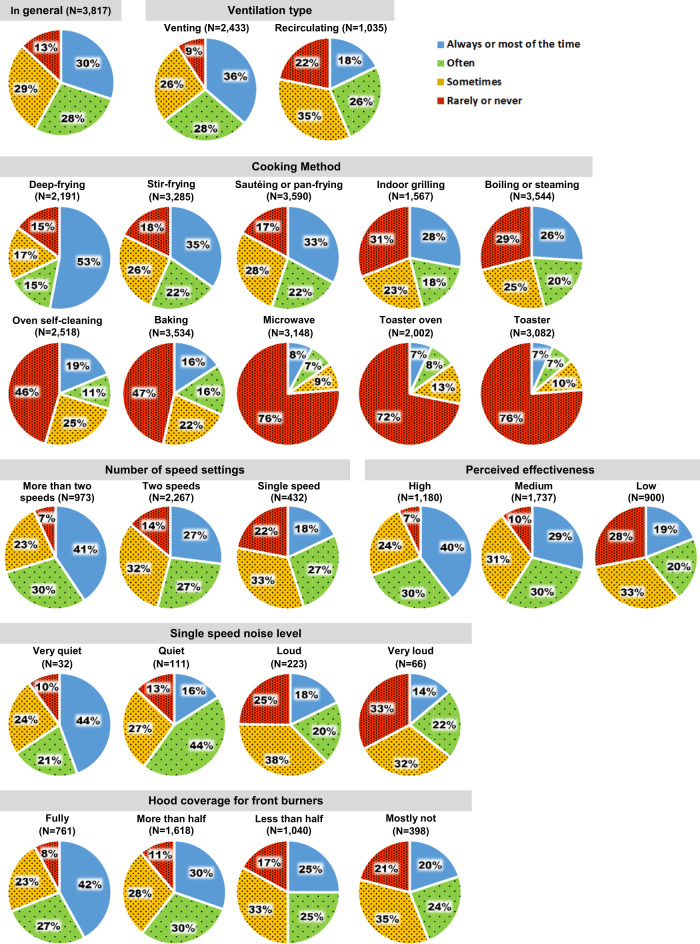
Use frequency of a cooking ventilation device for specific cooking activities and device characteristics. Results presented for 3817 survey responses who reported having a venting or recirculating device above or at the cooktop. Device use is categorized into four frequency categories: always or most of the time (blue), often (green), sometimes (yellow), and rarely or never (red).

The reported frequency of device use was associated with a number of factors, including cooking method (Cramer’s V = 0.28), ventilation type (Cramer’s V = 0.23), perceived effectiveness (Cramer’s V = 0.20), single speed noise level (Cramer’s V = 0.18), number of speed settings (Cramer’s V = 0.15), and hood coverage for front burners (Cramer’s V = 0.15). Importantly, these findings suggest that venting range hoods are more likely to be used on a regular basis than recirculating range hoods, with 64% of respondents who owned venting range hoods reported using them frequently (“often” or “most of the time”), compared to just 44% of those with recirculating hoods. Figure 3 shows how often the device is used during cooking in general and by influential factors. Respondents who reported not knowing how the ventilation device was used were excluded. The total number and standard error of responses by influential factors are presented in Table S1.

For respondents who had range hoods, OTR microwaves, or downdrafts, including both venting and recirculating devices, about 30% reported using them always or most of the time, 57% reported often or sometimes, and 13% reported rarely or never. Respondents reported using their devices most frequently for deep-frying; then stir-frying, sautéing or pan-frying (at similar levels); followed by indoor grilling, boiling, or steaming. Reported device use occurred least often when using a microwave, toaster oven, or toaster. Notably, 47% and 46% reported rarely or never using a device during oven baking and self-cleaning, respectively. Use of a device all or most of the time was much higher for devices that were venting vs. recirculating (36% vs. 18%), had more speed settings (41% for more than two, 18% for one setting), had a quiet fan if only one speed was available (44% for very quiet, 14% for very loud), provided more cooktop coverage (42% for full, 20% for mostly not), or was thought to remove cooking emissions effectively (40% for high, 19% for low). There was no significant difference in ventilation use between homes with electric versus gas stoves (*P* = 0.17).

The timing of usage relative to the start and end of cooking is important for venting devices. Households benefit most when using it during the entire cooking process. Of the respondents who had a venting device, only 6% indicated that they turned it on when turning on any cooktop burners and turned it off when turning off the last burner (Fig. S3). About 62% of respondents reported using the kitchen venting device only when cooking something that may produce smoke or odors, and 35% reported using it when sensing strong smoke or odors during cooking. Most of the respondents (78%) turned it off when they no longer need to remove smoke, odors, or heat, rather than continuing use until turning off all cooktop burners. Whether having a device or not, 64% of respondents indicated that, when possible, they open kitchen windows during cooking.

All range hoods need to have a mechanism to remove grease particles from the air pulled through the device. This is necessary to prevent combustible organic materials from reaching the motor assembly and duct system, and to reduce grease emissions into the kitchen. The most common form of grease collection is a metal screen; however, some devices use baffles or traps. The cleaning frequency for these devices depends on the design of the grease collection mechanism, the frequency of cooking with oils, and the amount of device use. Grease collected on a screen captures dust, lint, and other large particles creating a need to clean them as often as every 1–2 months with heavy use. A survey question asked, “If your ventilation device has metal grease screens, how often are they cleaned?” While 90% of respondents responded to this question, it is unclear how many of them had baffles or grease traps, and understood the question to refer to any grease capture equipment. With that caveat, it was reported that 17% of grease screens were cleaned every 1–2 months, 27% every 3–6 months, 36% at intervals longer than six months, and 11% were never cleaned. Although there was a strong association between the frequency of grease screen cleaning and range hood usage (Cramer’s V = 0.18), for those who cooked often (cooktop used at least five days a week) and reported using their range hood most of the time during cooking, only 29% and 33% reported cleaning the screens every 1–2 months and 3–6 months, respectively.

One-fourth of the survey respondents had a recirculating device. For this subgroup, 20% (5% of the overall sample) had a charcoal filter and 39% did not know whether their device had a charcoal filter. Twenty-two percent of charcoal filters changed color to indicate when replacement is needed; however, this feature did not lead to more frequent filter replacements in the past six months compared to filters that did not change color (*P* = 0.49). Figure S4 shows the percentage distribution of responses regarding filter replacement frequencies and reasons.

### Perspectives on behavioral recommendations in device use

Respondents who reported having a venting or recirculating device were asked about their knowledge of cooking pollutant hazards, willingness to try specific mitigation strategies (use the device more often, use cooktop back burners while using the device, and use higher speeds when needed) to reduce exposures, and reasons for not being compelled to follow the recommendations. Table 2 shows the willingness to adopt these strategies.

**Table 2:** Self-reported willingness of survey respondents to adopt ventilation strategies, after being informed through a survey prompt that cooking-related pollutants may present a hazard to human health.

Willingness to adopt the strategies	Ventilation device use strategies		
	Estimated population percentage (standard error)		
	Use the device more frequently during cooking	Cook more often on back burners while using the device	Use a higher speed when needed
Yes	44 (0.9)	44 (0.9)	45 (0.9)
Not sure	19 (0.7)	24 (0.8)	21 (0.8)
No	15 (0.7)	19 (0.7)	16 (0.7)
I am already doing so	23 (0.8)	13 (0.6)	18 (0.7)

About half of the respondents (52%) did not know that cooking generates unhealthy air pollutants and 39% did not know that cooking on back burners increases the efficiency of a range hood or OTR microwave because they are fully covered by the hood.

There were strong associations among the willingness to adopt the three recommendations (Cramer’s V = 0.28). Sixty-four percent of respondents indicated that they will consider adopting at least one recommendation, and 22% indicated that they will consider adopting all three recommendations. Twenty-four percent of respondents reported having already followed one of the three recommendations, and no respondents reported having followed all three recommendations already. The willingness to adopt the strategies was not statistically significantly different among respondents who reported having a venting or recirculating device.

Noise was the main reason for not using the ventilation device more often or not using a higher speed when needed. Low efficiency was the reason next to noise for not using the device more often. For respondents who reported not having a ventilation device, rental restrictions and no space in the kitchen were the most reported reasons for not installing one. There may have been other factors that prevented the installation of a ventilation device, such as an inability to vent to the outside, that were not surveyed.

## Discussion

The survey reported in this paper represents an important advance for understanding one of the most important residential indoor air quality hazards and the potential for mitigation through behavior change and equipment upgrades in Canadian homes. It may also support exposure assessment by providing population-representative data on cooking and ventilation activities. For example, Logue et al. [1] conducted a simulation-based assessment to estimate the impact of pollutant exposures from natural gas cooking burners in 6634 Southern California homes using data from published surveys and reports. Although our study was conducted in Canada, it is likely also broadly representative of the U.S. given the similarity in residential construction practices and cultural norms between the two countries.

This study has some limitations. Although recruitment was designed to capture representative samples of demographics and housing characteristics, both the pool and the response group could be biased in a number of ways. The sampling was restricted to respondents who were panel members, engaged in online surveys, and were English or French speakers. The performance of devices was evaluated based on respondents’ perceptions which can vary across individuals. Also, the validity and reliability of the self-reported data were not assessed by any on-site data collection. Some self-reported results likely have higher uncertainty, such as the ventilation equipment description (e.g., under-cabinet, wall-mounted, ceiling mounted, etc.) and the assessment of whether a ventilation device was ducted to the outside. The survey also did not include detailed questions about the presence and perceived effectiveness of continuous kitchen exhaust fans, which are often included in heat or energy recovery mechanical ventilation systems in both single and multiunit homes, as well as some high-rise apartment buildings.

Despite the limitations, the survey provides an overview of the characteristics of cooking activities and kitchen venting/recirculating device use in Canadian homes, and it identifies several issues. (1) Overall, devices are not used regularly by a substantial fraction of the population, especially when using ovens or toasters, and very few people operated their ventilation devices for the entire cooking process. (2) Only a small fraction of currently installed venting devices have all of the desired performance features (e.g., efficient, quiet operation, multiple speeds, full coverage of cooktop front burners). (3) Many people perceive that their devices have low effectiveness. (4) A large percentage of homes do not have either a venting device or a recirculating device with a charcoal filter that is replaced frequently. (5) There is a lack of awareness of cooking pollution hazards.

Thirty percent of respondents reported using their range hoods or OTR microwaves always or most of the time. This rate was higher than the usage recorded in time-activity diaries in IAQ studies conducted in 132 Edmonton and Halifax homes in Canada [47], where range hoods were used in 12% of the 2748 cooking activities. The inconsistency between the reported and actual use rates was also reported in other studies. A recent study conducted by Zhao et al. [48] found that for occupants who self-reported using their range hoods always or most of the time, the actual use rates were only 45% in 54 single-family houses and 36% in 17 low-income apartments in California. Willers et al. [58] reported a study of 74 homes in the Netherlands in which 91% of respondents indicated on a questionnaire that they often used their venting extractor fans during cooking, whereas only 69% reported doing so in time-activity diaries recorded during a field study.

Notwithstanding the bias between actual and self-reported device use, the self-reported device usage rate in this study was generally consistent with previous U.S. studies. In an online survey of cooking appliance usage in U.S. homes, 34% of 372 respondents reported using their range hoods always or most of the time during cooking [50]. In another U.S. survey on IAQ satisfaction and ventilation practices among residents of California homes built since 2002, 34% of 2516 respondents reported using their range hoods always or most of the time during cooking [53].

Kitchen ventilation device use could plausibly vary by cultural cooking and ventilation practices, by housing type, and by the interior separation of the kitchen; it is therefore interesting to compare the results of the Canadian survey to those from countries with different architectural (e.g., apartment residents) and cultural norms (e.g., Asian countries). A survey of 180 apartments in South Korea showed that residents did not actively ventilate the kitchen, and 16.7% of the residents did not apply any ventilation during cooking [54]. In a study estimating the contributions of indoor and outdoor sources to the exposures of PM_2.5_ and NO_2_, Hu et al. [51] conducted a survey on cooking, smoking, and air cleaning habits in 1103 urban households in China, and found that 30% of the high cooking frequency households (66% of total) used their range hoods frequently during cooking. Yin et al. [22] reported a much higher use rate, with 98% of the 120 Chinese high-rise apartment residences turned on the range hoods during cooking and 64% used the highest setting for the entire cooking process. The strong awareness of ventilation may be connected to the popularity of stir-frying as the major cooking style in the sampling residences [22].

In this study, over 70% of the respondents reported rarely or never using their devices while using microwaves, toaster ovens, or toasters, and about half of the respondents when using ovens. Several studies have shown that toasters and toaster ovens are common sources of PM_2.5_ and UFP exposures [47, 59]. Oven self-cleaning involves the use of high temperatures for an extended period of time, which can produce odors and a range of emissions, including CO, formaldehyde, acetaldehyde, and particulate matter, at high levels for both gas and electric ovens [9]. In addition, gas ovens produce large quantities of nitrogen oxides (NO, NO_2_), and potentially much higher levels of CO [9]. While cooking with a microwave typically releases much smaller quantities of particles [10, 60], substantial emissions can be produced when cooking popcorn in a pre-packaged foil-lined bag [61].

Using an over-the-range venting device during the cooking process can help to reduce exposure to pollutants generated by cooking and cooking burners, as it removes at least a portion of these pollutants [22, 38–47]. A study of California homes with gas cooking reported lower concentrations of NO_2_ and NO_X_ when range hoods were used [3]. Both capture efficiency and overall effectiveness can be further increased with higher airflow rates and cooking on back burners while using the device [39, 42–44, 46]. In the survey, 58% of the respondents prefer cooking on the cooktop front burners, while only 20% of them had a hood that fully covered the front burners. Singer et al. [43] demonstrated that pollutant capture efficiency can be reduced by 20–25% (absolute) when a range hood does not fully extend over the burners being used. In an experimental study by Sun et al. [46], at similar fan flow rates, a range hood with two inches less coverage in depth resulted in about a 100% increase in the cumulative exposure of UFP over the first hour after cooking.

The potential hazard of cooking emitted pollutants depends on exposure, which is a function of pollutant concentration and time, and the toxicity of the emitted pollutant mix. Although the cooking time could be short, the emitted air pollutants often mix throughout the house and substantially elevated levels may persist for hours as the contaminants are gradually removed by ventilation, deposition, and transport, or be removed more quickly by active air filtration [11, 44, 46, 47, 62, 63]. Newer homes, due to their tighter building envelopes, tend to have cooking pollutants that remain for longer periods compared to older homes of similar size and ventilation device settings [47]. In the survey, over 90% of respondents reported having a kitchen that opens to other parts of the home. The dispersion of cooking pollutants reduces pollutant concentrations by dilution, but it can also result in more people in the home being exposed. Transport can lead to exposure for occupants who are more vulnerable to the health effects of air pollution, such as young children, the elderly, those with respiratory or cardiovascular disease, and asthmatics. Therefore, the health risk from cooking emissions should not be considered for the cook only or in the kitchen only.

Twenty-five percent of homes in this survey had a recirculating range hood or OTR microwave. Use of these devices could pull some portion of cooking contaminants up from the cooktop area and distribute them out into the kitchen (or, depending on the height of the release point, back at the head of the cook); but many contain no mechanism to trap airborne contaminants. Some devices have charcoal filters, which are designed to absorb gaseous contaminants, including nitrogen dioxide, and may also remove some particles. Studies have shown that recirculating devices with new carbon filters can remove 20–50% of particles from the air moving through the device [64, 65]. In addition, the performance of charcoal filters will decrease with use. In a chamber study by Jacobs et al. [64], the NO_2_ removal efficiency of charcoal filters dropped from 60% when new to 20% after 19 days of cooking. The effectiveness of recirculating devices in reducing exposure in Canadian homes is likely to be low, as only 20% of respondents with recirculating devices reported that they have a carbon filter (with 39% unsure) and only 11% reported that the filter was replaced at least once within the past 12 months.

More attention needs to be given to residential kitchen ventilation. Education on cooking pollutants, health risks, and ventilation strategies can help promote higher device usage. For example, after knowing the impact of cooking on IAQ and the benefit of kitchen ventilation, more than half of the respondents indicated that they would consider following at least one recommendation. Of the respondents who reported not having a kitchen ventilation device, about one-third indicated that they would consider installing one. In addition to increasing public awareness, improving device performance is important. Increased availability and uptake of quieter and more efficient ventilation devices can be expected to lead to higher usage.

In conclusion, our study obtained population-representative data on the current availability and use of kitchen ventilation in Canadian homes, identified the influencing factors, and highlighted the need for increasing public education on cooking pollutants and ventilation strategies. The benefits of more frequent use and better effectiveness at removing contaminants should be synergistic. The much higher reported use rates of venting than recirculating devices and those with preferred performance features, including more speed settings, quieter operation, better cooktop burner coverage, and better perceived effectiveness, suggest great potential for higher-performing devices.

## Supplementary information

Supplemental Information

## Data Availability

The datasets generated during and/or analyzed during the current study are available from the corresponding author on reasonable request.
