# Supplementary material for: Cooking methods and kitchen ventilation availability, usage, perceived performance and potential in Canadian homes
Source: J Expo Sci Environ Epidemiol. 2023 Apr 15;33(3):439–47. doi: 10.1038/s41370-023-00543-z (PMC10234804; doi:10.1038/s41370-023-00543-z)
Supplement: Supplementary file 1 — Supplemental Information [file 41370_2023_543_MOESM1_ESM.pdf]

Supplemental Information

Cooking Methods and Kitchen Ventilation Availability, Usage, Perceived Performance and Potential in Canadian Homes

Liu Sun[1], Brett C. Singer[2]

[1] Air Sectors Assessment and Exposure Science Division, Water and Air Quality Bureau, Health Canada, Ottawa, Ontario, Canada

[2] Indoor Environment Group, Sustainable Energy and Environmental Systems Department, Energy Technologies Area, Lawrence Berkeley National Laboratory, Berkeley, California, USA

Corresponding author: liu.sun@hc-sc.gc.ca

Cooking and Kitchen Ventilation Survey Questionnaire

1. **Which category below includes your age?**
   a) 18-24
   b) 25-34
   c) 35-44
   d) 45-54
   e) 55-64
   f) 65 or older

2. **What is your gender?**
   a) Male
   b) Female
   c) Other (please specify) ___________

3. **Which province do you currently live in?**
   a) Quebec
   b) Ontario
   c) Manitoba
   d) Saskatchewan
   e) Alberta
   f) British Columbia
   g) Nova Scotia
   h) Newfoundland and Labrador
   i) Prince Edward Island
   j) New Brunswick

Section 1: Cooking and ventilation devices

4. **What is the fuel type of your primary cooking stove and oven?**

	**Electricity**	**Natural gas**	**Propane**	**Other, please specify**
**Stove**				
**Oven**				

5. **Do you have any of the following ventilation devices above (or at) the cooking stove?**

   a) Under-cabinet range hood 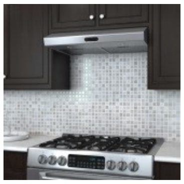

b) Wall-mounted range hood 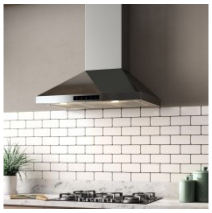

c) Ceiling-mounted range hood 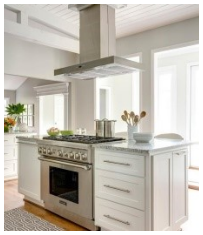

d) Over-the-Range microwave-hood combo 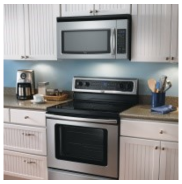

e) Downdraft exhaust 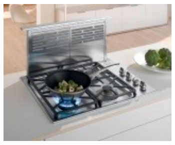

f) Other, please specify ______________

g) I do not have a ventilation device above (or at) the stove [if yes, then only answer Q6, Q13-16, Q28-30, Q36-42]

6. Other than the cooking area, does your kitchen have any additional ventilation devices?

a) Yes, please specify where it is located____

b) No

7. How many speed settings does your ventilation device have?

a) Single-speed

b) Two speeds

c) More than two speeds

d) Don't know [if yes, skip Q8]

8. How noisy is your ventilation device on various fan speed settings?

[If Q7=single-speed]

	Very quiet	Quiet	Loud	Very loud
Single-speed				

If [Q7=two speeds]

	Very quiet	Quiet	Loud	Very loud
Low speed				

High speed				

If [Q7=more than two speeds]

	Very quiet	Quiet	Loud	Very loud
Lowest speed				
Medium speeds				
Highest speed				

9. Is your ventilation device ducted to the outside?

a) Ducted to the outside

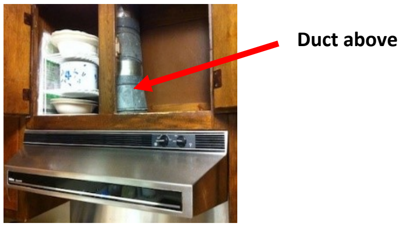

b) Not ducted to the outside, blowing air back into the kitchen

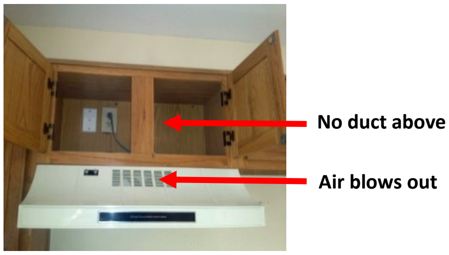

c) Don't know

10. To what extent are the stove front burners covered by your ventilation device?

a) Fully
b) More than half
c) Less than half
d) Mostly not

11. How effective is your ventilation device in removing the following emissions from cooking?

	None is removed	Some are removed	Most are removed	All are removed
Smoke				
Odor				
Heat				
Moisture				

Grease				

12. Based on your experience, what aspects of your ventilation device, if any, need to be improved? Select all that apply.

a) Smoke removal
b) Odor removal
c) Heat removal
d) Grease capture
e) Noise
f) More coverage of stove front burners
g) Easier to clean grease screens
h) Easier to purchase carbon filters
i) Easier to change carbon filters
j) Other (please specify) _____________
k) No improvement needed

Section 2: Cooking behavior

13. How many days a week do you use the following cooking appliances?

	Every day (7 days)	5-6 days	3-4 days	1-2 days	I typically do not use it
Stovetop					
Oven					
Microwave					
Toaster					
Toaster oven					
Indoor grill					
Air fryer					
Rice cooker					
Slow cooker					
Stand-alone pressure cooker					
Stovetop pressure cooker					

14. For the following meals, what are your most frequent cooking styles? Select up to 3 options per meal.

	Deep-frying	Stir-frying	Sautéing or pan-frying	Baking	Boiling or steaming	Grilling	Microwave	Toasting	Other, please specify	Do not cook for this meal
Breakfast										

Lunch										
Dinner										

15. Which stove burners do you use most often?

a) Front burners
b) Back burners
c) Use both equally

16. In which season do you cook using a stove or oven the most?

a) Spring
b) Summer
c) Fall
d) Winter
e) Same in all seasons

Section 3: Ventilation device use behavior

17. In general, how often do you use your ventilation device during cooking?

a) Always or most of the time
b) Often
c) Sometimes
d) Rarely or never
e) Don't know

18. How often is your ventilation device turned on while:

	Always or most of the time	Often	Sometimes	Rarely or never	Don't know	Do not cook this way
Deep-frying						
Stir-frying						
Sautéing or pan-frying						
Oven baking						
Indoor grilling						
Boiling or steaming						
Using the toaster						
Using the toaster oven						
Using the microwave						
Oven self- cleaning						

19. When you use your ventilation device, what are the main reasons? Select all that apply.

a) Remove smoke

b) Remove odors
c) Remove moisture
d) Remove heat
e) Capture grease
f) Remove air pollutants emitted by cooking
g) As a general ventilation method for the kitchen
h) Never use it [skip Q20-22]
i) Other, please specify _______________

20. When do you usually turn the ventilation device on while cooking? Check all that apply

a) When turning on any stove burner
b) When starting to cook something that can produce smoke or odors
c) Only when I sense strong smoke, odors, or heat
d) Only when cooking causes uncomfortable air conditions

21. When do you usually turn the ventilation device off while cooking?

a) When turning off the last burner
b) When no longer needed to reduce smoke, odors, or heat
c) Other, please specify ____________

22. When using the ventilation device, which fan speed do you use most often?

[If Q7=two speeds]
a) Low
b) High
c) Varies depending on what I am cooking

[If Q7= More than two speeds]
a) Lowest
b) Middle
c) Highest
d) Varies depending on what I am cooking

23. If your ventilation device has metal grease screens, how often are they cleaned?

a) Every 1-2 months
b) Every 3-6 months
c) Every 7-12 months
d) More than 1 year or longer
e) Never
f) no grease screens

24. Do you have charcoal or carbon filters installed in your ventilation device?

a) Yes
b) No [skip Q25-26]
c) Don't know [skip Q25-26]

25. How often do you replace charcoal or carbon filters?

a) Every 1-2 months
b) Every 3-6 months
c) Every 7-12 months
d) More than 1 year or longer
e) Haven't changed so far [please specify how many years the range hood has been used_____ [skip Q26]

26. How do you decide to change the charcoal or carbon filters?

a) When the performance declines
b) The filters have an indicator (e.g. changing colors or patterns)
c) Other, please specify______
d) Not sure

27. Other than using a range hood, do you clean the air of your home in other ways while cooking? Check all that apply.

a) Opening kitchen windows, when possible
b) Opening windows of other rooms, when possible
c) Running bathroom fans
d) Running a central heating or cooling fan
e) Running a portable air cleaner
f) Other (please specify) ____________
g) No

28. Cooking can generate unhealthy air pollutants from heating oil, fat, and other food ingredients, especially at high temperatures. Have you heard of this before?

a) Yes
b) No

29. Running a range hood not only reduces cooking odors or smoke but also reduces the air pollutants generated by cooking. Knowing this, will you use the range hood more often than you have in the past?

a) Yes [skip Q30]
b) No
c) Not sure
d) I always use the range hood during cooking [skip Q30]

30. Select the reason(s) for not using the range hood more often. Check all that apply.

a) Noisy
b) Not effective
c) Uses lots of energy
d) I open windows instead
e) I use an air cleaner instead
f) Air pollutants emitted by cooking are not dangerous to my health
g) Other (please specify) __________
[Add the following three options only if Q5= does not own a range hood]
h) Cost too much
i) No space in the kitchen for a range hood
j) Rental restrictions

31. Cooking on BACK burners can increase the efficiency of a range hood because they are fully covered by the hood. Have you heard of this before?

a) Yes
b) No

32. Knowing that the range hood works better with the back burners, will you cook more often on the back burners (with a range hood running) than you have in the past?

a) Yes [skip Q33]
b) No
c) Not sure
d) I already cook often on the backburners [skip Q33]

33. Select the reason(s) for not using the back burners more often. Check all that apply.

a) Not convenient
b) Burner size restrictions (e.g. only front burners provide the size that I use often).
c) My range hood is not located above the range.
d) Not necessary, my range hood covers all the stove burners
e) I open windows instead
f) I use an air cleaner instead
g) Air pollutants emitted by cooking are not dangerous to my health
h) Other, please specify ___________

34. Running a range hood at higher speeds can greatly reduce cooking-related pollutants. Knowing that, will you use a higher speed more often when frying, sautéing, or grilling?

a) Yes [skip Q35]
b) No

c) Not sure
d) I always use a higher speed when frying, sautéing, or grilling [skip Q35]

35. Select the reason(s) for not using a higher speed more often. Check all that apply.

a) It is the same at either speed of my range hood
b) Too noisy at a higher speed
c) Uses lots of energy
a) I open windows instead
b) I use an air cleaner instead
c) Air pollutants emitted by cooking are not dangerous to my health
d) Other, please specify ____________

Section 4: Dwelling information

36. Which category below describes your home?

a) Detached house
b) Attached house (such as townhouse, row house, or semi-detached house)
c) Apartment
d) Mobile home
e) Other, please specify _______
f) Don't know

37. Do you own your home?

a) Yes
b) No (e.g. rented)

38. In what year was your home built?

a) Before 1900
b) 1900-1940
c) 1941-1960
d) 1961-1980
e) 1981-2000
f) After 2000
g) Don't know

39. What is the size of your home (excluding the basement)?

a) <1000 ft^2 (93 m^2)
b) 1001-2000 ft^2 (94-186 m^2)
c) 2001-3000 ft^2 (187-279 m^2)
d) >3000 ft^2 (280 m^2)
e) Don't know

40. How is your kitchen connected to other parts of your home?

a) Part of the room that includes the dining or family room (open-concept kitchen)
b) Separate room with open doorways connecting to other rooms
c) Separate room with a door(s) that can be closed
d) Other (please specify) _________
e) Don't know

41. How many people in each age category currently live in your home?

	0	1	2	3	4 and above
Young children (0-5 years old)					
Children (6-14 years old)					
Youth (15-24 years old)					
Adults (25 – 64 years old)					
Seniors (65 years and over)					

42. Which category below describes your 2019 household income before taxes?

a) $19,999 and under
b) $20,000 to $39,999
c) $40,000 to $59,999
d) $60,000 to $99,999
e) $100,000 to $149,999
f) $150,000 and over
g) Prefer not to answer

Table S1. Percentage distribution of responses regarding device use frequency by influential factors.

Influential factors	Group (N)	Device use frequency<br>Adjusted population percentage (standard error)			
		Always or most of the time	Often	Sometimes	Rarely or never
**Ventilation type**	Venting (2,433)	36 (1.1)	28 (1.1)	26 (1.0)	9.1 (0.6)
	Recirculating (1,035)	18 (1.4)	26 (1.6)	35 (1.7)	22 (1.5)
**Cooking method**	Deep-frying (2,191)	53 (1.2)	15 (0.9)	17 (0.9)	15 (0.9)
	Stir-frying (3,285)	35 (1.0)	22 (0.8)	26 (0.9)	18 (0.8)
	Sautéing or pan-frying (3,590)	33 (0.9)	22 (0.8)	28 (0.9)	17 (0.7)
	Indoor grilling (1,567)	28 (1.3)	18 (1.1)	23 (1.3)	31 (1.4)
	Boiling or steaming (3,544)	26 (0.9)	20 (0.8)	25 (0.8)	29 (0.9)
	Oven self-cleaning (2,518)	19 (0.9)	11 (0.7)	25 (1.0)	46 (1.1)
	Oven baking (3,534)	16 (0.7)	16 (0.7)	22 (0.8)	47 (1.0)
	Microwave (3,148)	7.7(0.5)	7.4 (0.5)	8.5 (0.6)	76 (0.9)
	Toaster oven (2,002)	6.9 (0.6)	8.3 (0.7)	13 (0.9)	72 (1.1)
	Toaster (3,082)	6.7 (0.5)	7.1 (0.5)	9.9 (0.6)	76 (0.9)
**Speed setting**	More than two speeds (973)	41 (1.8)	30 (1.7)	23 (1.5)	6.5 (0.9)
	Two speeds (2,267)	27 (1.1)	27 (1.1)	32 (1.1)	14 (0.8)
	Single speed (432)	18 (2.1)	27 (2.6)	33 (2.7)	22 (2.2)
**Single speed noise level**	Very quiet (32)	44 (9.3)	21 (8.2)	24 (8.0)	10 (5.7)
	Quiet (111)	16 (4.0)	44 (5.6)	27 (4.7)	13 (3.1)
	Loud (223)	18 (2.9)	20 (3.2)	38 (3.9)	25 (3.3)
	Very loud (66)	14 (4.4)	22 (6.9)	32 (7.1)	33 (6.7)
**Hood coverage for front burners**	Fully (761)	42 (2.1)	27 (1.9)	23 (1.7)	7.6 (1.1)
	More than half (1,618)	30 (1.3)	30 (1.3)	28 (1.3)	11 (0.8)
	Less than half (1,040)	25 (1.6)	25 (1.6)	33 (1.7)	17 (1.3)
	Mostly not (398)	20 (2.3)	24 (2.6)	35 (2.8)	21 (2.4)
**User-perceived effectiveness[†]**	High (1,180)	40 (1.6)	30 (1.6)	24 (1.4)	6.6 (0.7)
	Medium (1,737)	29 (1.3)	30 (1.3)	31 (1.3)	10 (0.8)
	Low (900)	19 (1.5)	20 (1.6)	33 (1.8)	28 (1.7)

[†] device effectiveness was summarized based on the reported effectiveness in removing smoke, odor, moisture, and heat removal, as well as grease capture.

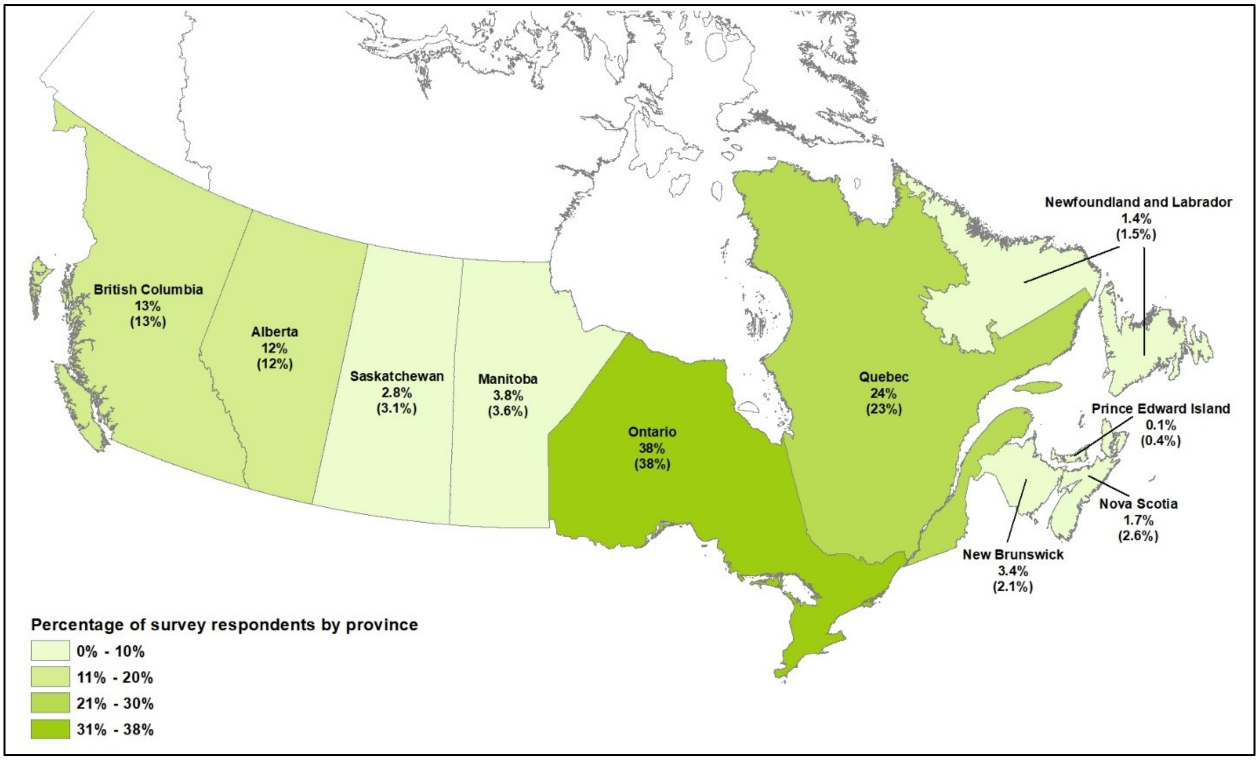

Figure S1. Percentage of survey respondents (N=4,500) and the 2016 Canadian Census population (in brackets) by province.

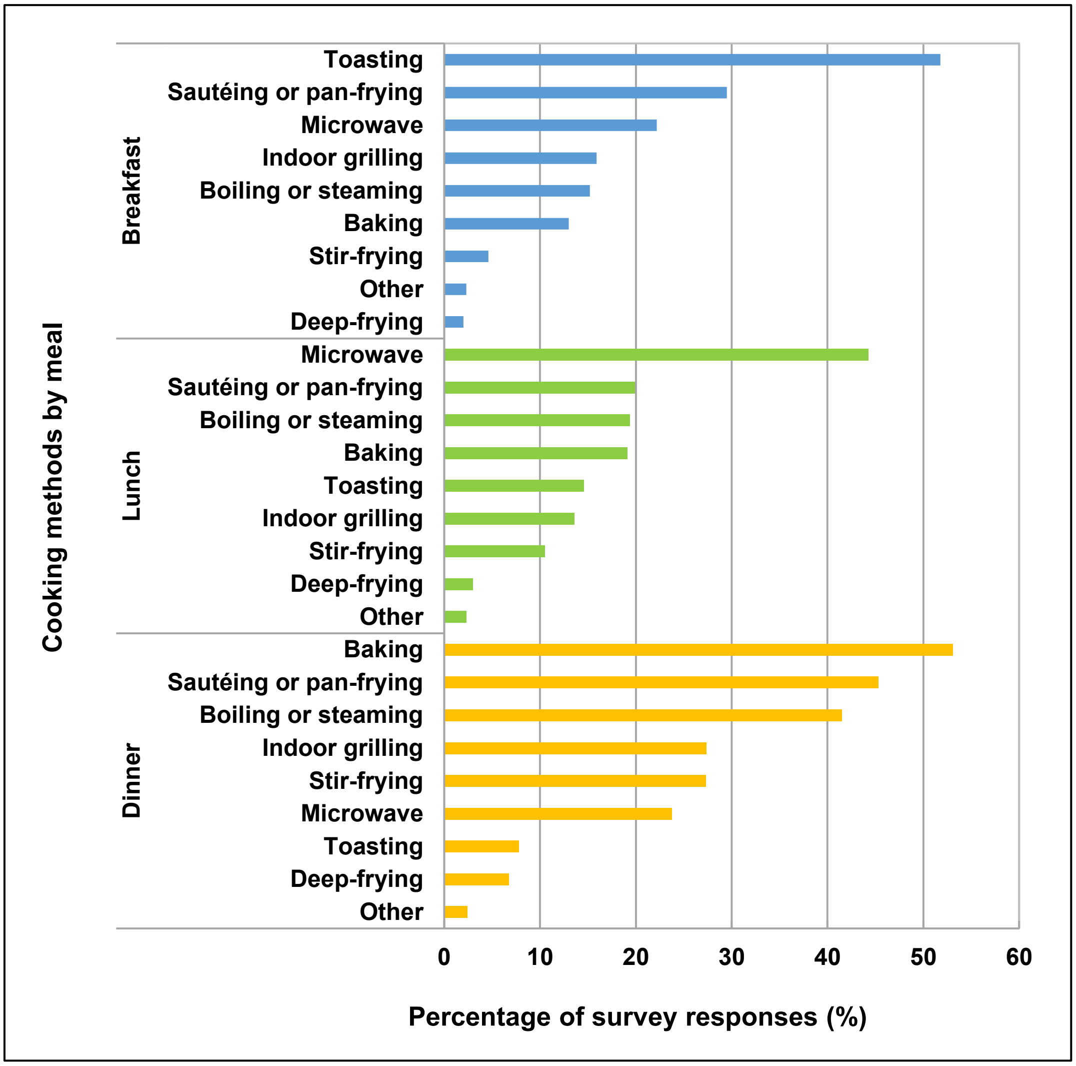

Figure S2. Most commonly used cooking methods for each meal among survey respondents (N=4,500).

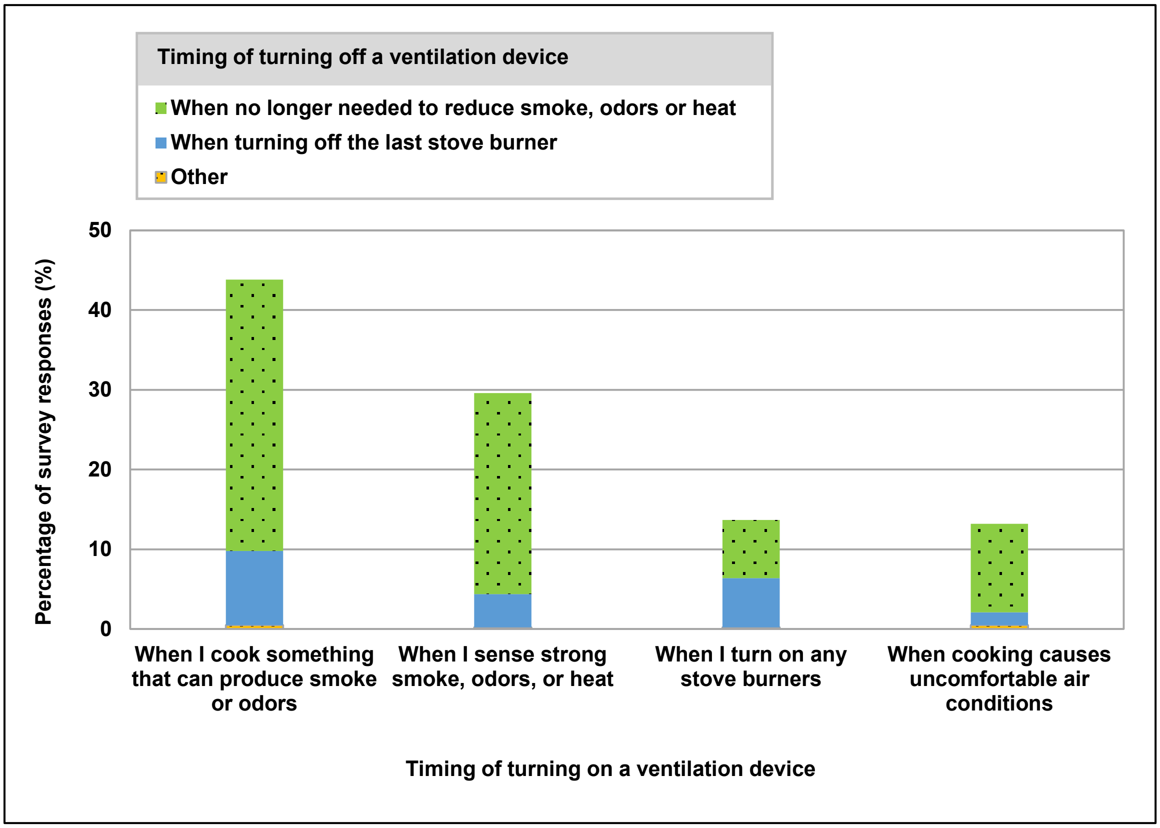

Figure S3. Percentage distribution of survey responses (N=3,890) regarding the timing of turning on and off a ventilation device during cooking. Respondents (N=610) who reported not having a venting or recirculating ventilation device were excluded.

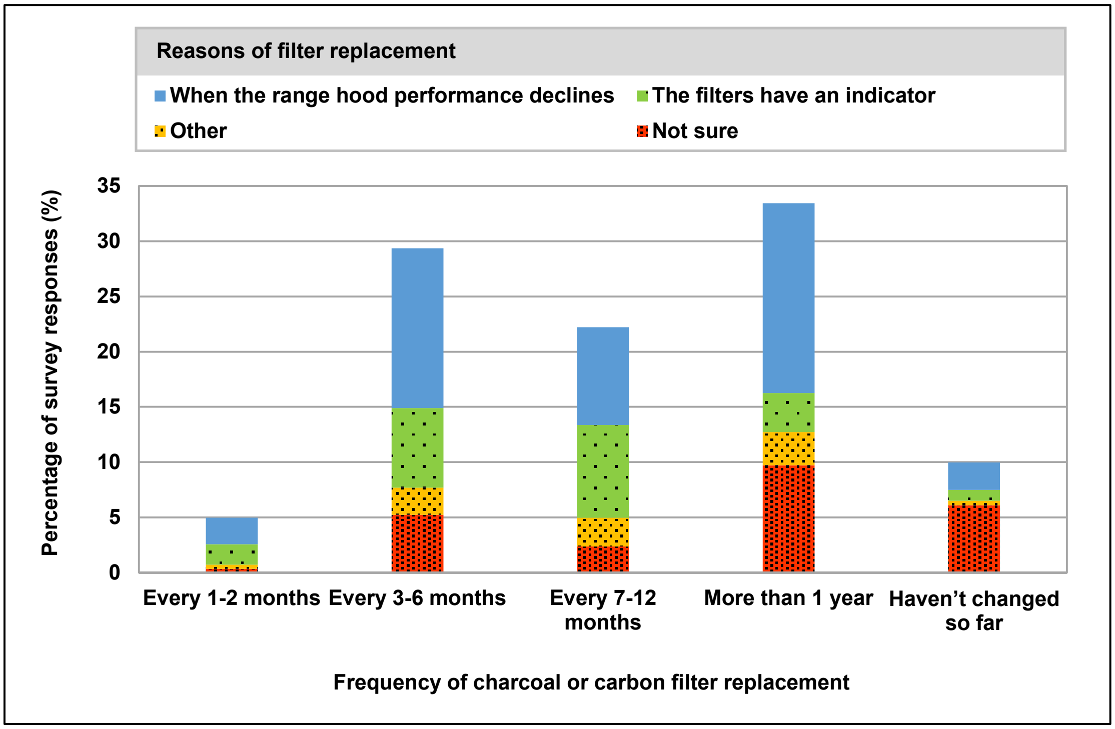

Figure S4. Percentage distribution of survey responses (N=211) regarding charcoal or carbon filter replacement frequencies and reasons.
